# Non-BRCA1/BRCA2 high-risk familial breast cancers are not associated with a high prevalence of BRCAness

**DOI:** 10.1186/s13058-023-01655-y

**Published:** 2023-06-14

**Authors:** Lars v. B. Andersen, Martin J. Larsen, Helen Davies, Andrea Degasperi, Henriette Roed Nielsen, Louise A. Jensen, Lone Kroeldrup, Anne-Marie Gerdes, Anne-Vibeke Lænkholm, Torben A. Kruse, Serena Nik-Zainal, Mads Thomassen

**Affiliations:** 1 Department of Clinical Genetics, Odense University Hospital, Odense, Denmark; 2 Clinical Genome Center, Department of Clinical Research, University of Southern Denmark, Odense, Denmark; 3 Hutchison Research Centre, Early Cancer Institute, University of Cambridge, Cambridge Biomedical Campus, Cambridge, CB2 0XZ UK; 4 Academic Laboratory of Medical Genetics, Lv 6 Addenbrooke’s Treatment Centre, Addenbrooke’s Hospital, Cambridge, CB2 0QQ UK; 5 European Sperm Bank, Copenhagen, Denmark; 6 Department of Clinical Genetics, Copenhagen University Hospital Rigshospitalet, Copenhagen, Denmark; 7 Department of Surgical Pathology, Zealand University Hospital, 4000 Roskilde, Denmark

**Keywords:** Breast cancer, Hereditary breast cancer, Non-BRCA1/BRCA2, Whole genome sequencing, Mutational signatures, HRDetect, BRCAness

## Abstract

**Background:**

Familial breast cancer is in most cases unexplained due to the lack of identifiable pathogenic variants in the *BRCA1* and *BRCA2* genes. The somatic mutational landscape and in particular the extent of BRCA-like tumour features (BRCAness) in these familial breast cancers where germline *BRCA1* or *BRCA2* mutations have not been identified is to a large extent unknown.

**Methods:**

We performed whole-genome sequencing on matched tumour and normal samples from high-risk non-*BRCA1/BRCA2* breast cancer families to understand the germline and somatic mutational landscape and mutational signatures. We measured BRCAness using HRDetect. As a comparator, we also analysed samples from *BRCA1* and *BRCA2* germline mutation carriers.

**Results:**

We noted for non-*BRCA1/BRCA2* tumours, only a small proportion displayed high HRDetect scores and were characterized by concomitant promoter hypermethylation or in one case a RAD51D splice variant previously reported as having unknown significance to potentially explain their BRCAness. Another small proportion showed no features of BRCAness but had mutationally active tumours. The remaining tumours lacked features of BRCAness and were mutationally quiescent.

**Conclusions:**

A limited fraction of high-risk familial non-*BRCA1/BRCA2* breast cancer patients is expected to benefit from treatment strategies against homologue repair deficient cancer cells.

**Supplementary Information:**

The online version contains supplementary material available at 10.1186/s13058-023-01655-y.

## Background

Approximately 5–10% of all breast cancer cases are familial [1–3]; however, less than 17–28% are attributed to inherited mutations in the *BRCA1* and *BRCA2* susceptibility genes [4–6]. This challenges clinical genetic counselling of families with a strong history of breast cancer without identified germline mutations in *BRCA1* and *BRCA2* (hereafter referred to as non-*BRCA1/BRCA2* high-risk families). Recent studies using whole-genome sequencing (WGS) have resulted in a comprehensive landscape of somatic mutations revealing the mutational processes that have left specific mutational signatures in the tumours. These signatures may be predictive of treatment response. HRDetect is a robust prediction model incorporating mutational signatures, HRD-index, and deletion of microhomology [7]. HRDetect has been shown to be predictive among non-*BRCA1/BRCA2* patients for response to platinum-based chemotherapy [8]. A recent clinical trial demonstrated that breast cancer patients with germline mutations in *BRCA1* or *BRCA2* benefit from Poly(adenosine diphosphate–ribose) polymerase (PARP) inhibitor treatment [9]. Non-*BRCA1/BRCA2* patients with a high HRDetect score may potentially also benefit from this treatment.

Although the somatic mutational landscape, molecular signatures, and HRDetect are well-established in unselected breast cancer, studies of these features among non-*BRCA1/BRCA2* high-risk familial breast cancers are limited [10]. In this study, we therefore applied WGS to a new cohort of these patients and analysed molecular subtypes based on HRDetect, mutational load, and molecular signatures.

## Methods

We performed WGS of flash frozen primary breast samples and matched normal blood samples from 23 breast cancer patients from high-risk breast and ovarian cancer families screened negative for mutations in *BRCA1* and *BRCA2* together with seven patients carrying a pathogenic *BRCA1* or *BRCA2* variant (Additional file 1: Figure S1c, Additional files 5, 6: Table S1–S2).

We identified somatic substitutions, insertions and deletions (indels), and rearrangements in our cohort as previously described [11]. Then we fitted the catalogues of somatic mutations to the previously identified substitution and rearrangement signatures in breast cancer using a mathematical model [12]. Unsupervised hierarchical clustering was applied to stratify the tumours based on the somatic mutational signatures. Moreover, we applied the HRDetect model to identify *BRCA1/BRCA2*-deficient tumours (BRCAness) driving tumourigenesis by defective homologous recombination [7]. We also analysed germline variants for potential causal variants in other known cancer predisposing genes and a polygenic risk score (PRS) based on 313 SNPs [13].

A detailed description of the included patients and all performed analyses is provided in the Additional method section.

## Results

**Fig. 1:**
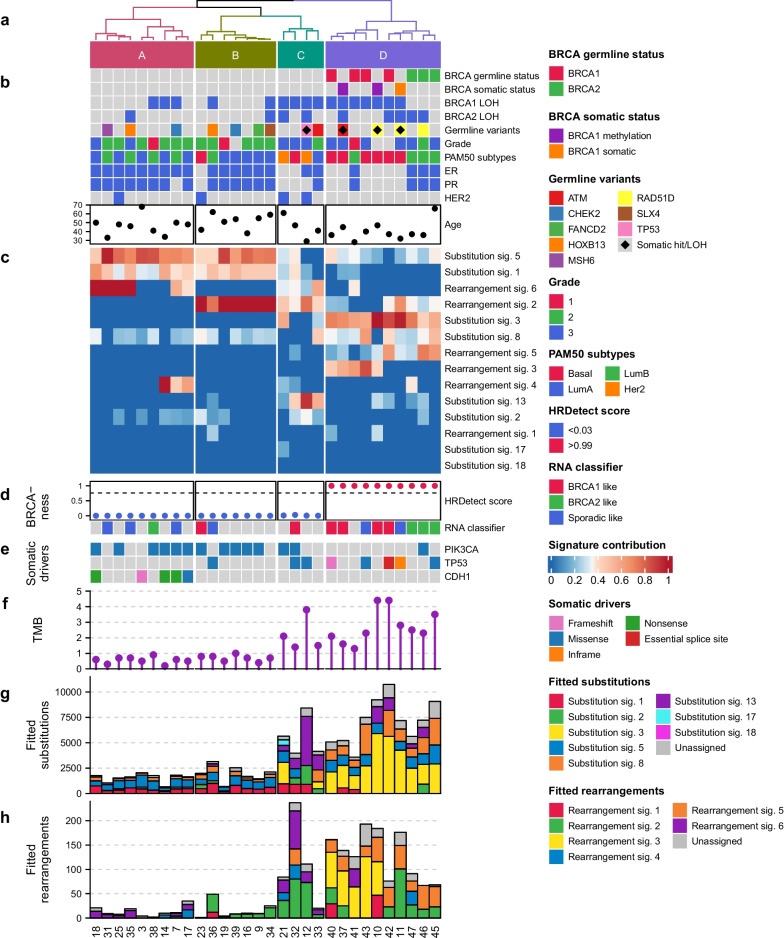
Unsupervised hierarchical clustering based on proportions of mutational signatures in each tumour. **a** Unsupervised hierarchical clustering on substitution and rearrangement signatures revealing four main clusters. **b** Clinical and mutational annotation for each sample. **c** Heatmap of the normalised contribution of substitution and rearrangement signatures identified in the cohort. **d** BRCAness predictions: HRDetect prediction score (scores above 0.7 considered BRCAness) and RNA classifier predictions. **e** Somatic substitution and indel driver mutations are present in more than three samples. **f** Tumour mutational burden of somatic mutations per Mb in the coding region. **g** Number of substitutions fitted to mutational signatures for each tumour. **h** Number of rearrangements fitted to mutational signatures for each tumour

The somatic mutational landscape of high-risk familial non-*BRCA1/BRCA2* tumours revealed distinct levels of genomic instability among the tumours (Fig. 1e-h, Additional file 2, 3: Figure S2-–S3, Additional file 7: Table S3). For identification of *BRCA1/BRCA2*-deficiency, we applied HRDetect to our cohort. This resulted in a very strong separation of tumours with all *BRCA1* and *BRCA2* positive tumours having a HRDetect score of > 0.99 (Fig. 1d). For further characterization of molecular features, we identified mutational signatures (Fig. 1c and Additional file 4: Figure S4). Clustering based on these signatures supported that *BRCA1/BRCA2*-mutated tumours are clearly separated from non-*BRCA1/BRCA2* tumours (Fig. 1a and c). The separation from the non-BRCA1/BRCA2 tumours is mainly driven by homologue repair deficiency (HRD), i.e. the HRD-associated substitution signature (SBS) 3, where a higher level is detected in the *BRCA1/BRCA2*-mutated tumours. When observing the expression patterns, SBS 3 is somewhat co-expressed with SBS 8, a signature still considered to have unknown aetiology. One study has suggested SBS 8 is characterised by replication errors [14]. However, this is not clear since the *BRCA1/BRCA2* genes are mainly associated with double-stranded DNA repair.

Three non-*BRCA1/BRCA2* tumours also had a HRDetect score of > 0.99 and were classified as having *BRCA1/BRCA2*-deficient tumours. These tumours clustered together with the *BRCA1/BRCA2* tumours (cluster D in Fig. 1, Additional file 8: Table S4). All three tumours had *BRCA1*-like characteristics, i.e. were triple negative (negative estrogen/progesterone receptor (ER/PR) and HER2 receptor status normal), basal-like subtype and had *BRCA1* loss of heterozygosity (LOH). Promoter hypermethylation of *BRCA1* likely explains *BRCA*-deficiency in two of these tumours (tumour 10 and 37). In addition, tumour 10 harboured a likely pathogenic germline *RAD51D* splice variant, c.738 + 1G > A, expected to contribute to BRCAness in this tumour since inactivation of this gene is known to result in HRD [15]. The last tumour (tumour 11) with a high HRDetect score contained both a somatic BRCA1 missense variant and a germline *RAD51D* variant of unknown significance. The *BRCA1* missense variant c.3668 T > A, p.(Leu1223His) is located in exon 11 that, although containing more than half of the coding region of *BRCA1*, does not contain reported pathogenic germline missense mutations. Furthermore, a second hit, i.e. LOH, was not observed. The *RAD51D* variant c.202G > A, p.(Gly68Ser) in contrast displayed LOH in this tumour (Additional file 9: Table S5). This variant has previously been reported to inactivate an ESE element and cause complete loss of full-length transcript. However, since 32% of the transcripts had an in-frame deletion of 12 amino acids the variant was classified as having unknown significance [16]. Our results strongly indicate the variant to be deleterious for the protein and therefore likely pathogenic. This variant was also identified in another tumour (tumour 46, Fig. 1). LOH was not detected; however, bi-allelic inactivation of BRCA2 is likely to explain HRD in this tumour.

The clustering also revealed a group of tumours with high mutational burden and high level of the APOBEC associated SBS 13 but with a low HRDetect score (cluster C in Fig. 1). These tumours more frequently harboured *BRCA1/BRCA2* LOH, *TP53* mutations, and had negative ER/PR and positive HER2 status. Finally, the clustering revealed two clusters of non-*BRCA1/BRCA2* tumours with distinct molecular profiles not related to known molecular subtypes (clusters A and B in Fig. 1). These tumours all had a very low mutational burden, low HRDetect scores (0.03 or less) and a higher frequency of somatic *PIK3CA* mutations and a lower frequency of *TP53* mutations compared to tumours with a high mutational burden (Fig. 1, Additional file 2: Figure S2, Additional file 8, 9: Table S4-S5). Tumours in cluster A were mainly described by SBS 1 and 5, lack of rearrangement signature (RS) 2, were mainly luminal (Lum) A or B, and commonly had *CDH1* mutations. Tumours in cluster B were primarily defined by SBS 1 and 5, RS 2, and were mainly LumA. Cluster B clustered close to cluster C containing tumours with high mutational burden, as these tumours also had high proportions of RS 2 (Fig. 1).

We integrated the results from our previously published RNA classifier to classify basal-like tumours as either *BRCA1*-like or non-*BRCA1*-like, and LumB-subtype tumours as either *BRCA2*-like or non-*BRCA2*-like. [17, 18]. The RNA classifier performed slightly differently from HRDetect; one *BRCA1* positive tumour was not classified as *BRCA1*-Like. The tumour (tumour 11) with a somatic *BRCA1* missense VUS and *RAD51D* c.202G > A variant were not classified as *BRCA1*-Like supporting that this tumour is driven by *RAD51D* and not *BRCA1* (Fig. 1d). In contrast, the tumour with bi-allelic *BRCA2* inactivation and *RAD51D* c.202G > A (tumour 46) was classified as *BRCA2*-like supporting the inactivation of *BRCA2* as the driver of this cancer. Among non-*BRCA1/BRCA2* tumours, the RNA classifier identified 5/23 (22%) tumours with BRCAness compared to HRDetect predicting 3/23 (13%) tumours as having BRCAness.

**Fig. 2:**
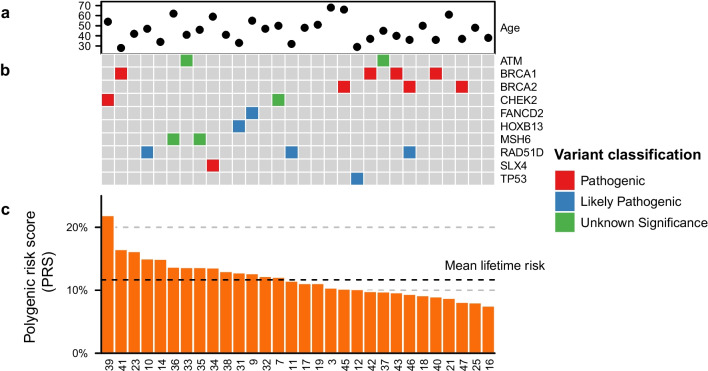
Germline variants and PRS predicted lifetime risk (20–80 years) of breast cancer in high-risk familial breast cancer patients. Samples are ordered according to PRS score. **a** Age of diagnosis. **b** Germline variants possibly contributing to increased breast cancer family risk. Potential breast cancer predisposing genes were included if they are reported pathogenic/likely pathogenic in ClinVar. VUS were included for known moderate and high-risk genes. **c** Predicted lifetime risk of breast cancer by PRS score. The dashed line represents the mean lifetime risk (0–79 years) of breast cancer in the Danish population of 11.7% [51]

Germline variants could only explain a few cases of familial aggregation. We identified rare germline variants in the *FANCD2*, *RAD51D*, *TP53*, *SLX4*, *MSH6* and *CHEK2* genes of which *RAD51D, TP53* and *CHEK2* are likely to contribute to familial aggregation (Fig. 2, Additional file 10: Table S6). Furthermore, we applied the PRS [13] incorporated in BOADICEA [19] to obtain the estimated lifetime risk and the combined risk with family history. The patient carrying the pathogenic *CHEK2* mutation had a high PRS score resulting in an estimated lifetime risk of 57%, where PRS contributed with 22% (patient 39, Fig. 2). This was further supported by the bilateral breast cancer of the patient and family history of multiple breast cancer cases. The remaining patients showed little or negative effect contributed by the PRS (Fig. 2, Additional file 1: Figure S1, Additional file 5: Table S1).

## Discussion

The finding of a low fraction of high-risk non-*BRCA1/BRCA2* familial breast cancers with high HRDetect scores is noteworthy, as one might expect non-*BRCA1/BRCA2* high-risk familial breast tumours to have a higher frequency of high HRDetect scores similar to tumours from families with mutations in these high-penetrant genes. Especially, considering that our families are selected based on a combination of multiple breast cancer cases, early onset of breast cancer, and ovarian cancer in the families, criteria that makes them very similar to the families with *BRCA1/BRCA2* mutations. The majority of the tumours are ER-positive, a molecular subtype mostly associated with HR-proficiency. However, this might not be the case for hereditary cases, with *BRCA2*-related cancer as a prominent example where tumours most often are ER-positive but *BRCA1/BRCA2*-deficient [11, 20]. Large panel sequencing studies of HRD related genes have identified a low fraction of familial cases explained by non-*BRCA1/BRCA2* germline variants in HRD-genes [21, 22], and in a study by Matis et al. [23], it was concluded that the likelihood of associating more HRD-genes with familial breast cancer is low. Our results support these findings and thereby indicate that other mechanisms such as mutations in non-coding regions leading to HRD are not likely to play a major role. The results of low HRDetect scores in our high-risk non-*BRCA1/BRCA2* familial breast tumours are also supported by an independent method, our RNA-classifier, although a few tumours with low HRDetect scores were *BRCA1*- or *BRCA2*-like with this profile. Further studies are required to investigate if BRCAness at the RNA level exists due to other mechanisms than mutational patterns. Molecular signature analysis has previously been shown to be useful in the characterisation of variants [24], and the identification of high HRDetect and LOH in the tumour carrying the spliciogenic variant c.202G > A and LOH at the RAD51D locus illustrate this.

In studies conducted on several complex diseases, including breast cancer, a low PRS score has been found to be associated with the finding of rare, pathogenic variants [25, 26]. This is supported in our study where most of the variants classified as pathogenic are also found to have a low contribution of PRS to the lifetime risk of developing breast cancer.

To the best of our knowledge, only one other study by Nones et al. [10] has applied WGS to characterise familial breast cancer. Their study included 30 familial non-*BRCA1/BRCA2* breast tumours. The study identified 4 (13%) non-*BRCA1/BRCA2* tumours with high *BRCA1/BRCA2* HRDetect scores that could not be explained by promotor methylation or somatic mutations. This is a slightly higher fraction than in our material where only one such tumour (4%) was identified and even that harboured a somatic VUS in *BRCA1* and a germline splice variant in and LOH in *RAD51D*. Nones et al. also identified a cluster of silent tumours with RS 2 like we did (cluster B in Fig. 1). However, they also found a large cluster of 13/30 (43%) tumours with high contribution of RS 4 affecting known driver genes. We only identified 3/23 (13%) tumours having high proportions of RS 4. The differences between the two studies might to some extent be explained by the approaches used to identify mutational signatures. Nones et al. extracted novel signatures and correlated these to the known COSMIC signatures, whereas we opted to fit the catalogue of somatic mutations directly to the known breast cancer signatures as this is more suitable for small sample sizes [12, 27, 28]. Their study population of non-*BRCA1/BRCA2* tumours had a similar sample size to our study. However, both studies are statistically underpowered to draw significant conclusions. Nevertheless, their findings of limited BRCAness among cases with suspicion of hereditary breast cancer are very similar to ours. The combined results from the two studies strongly indicate a low frequency of BRCAness among non-*BRCA1/BRCA2* familial breast cancer patients with no identified variants in other HRD genes.

## Conclusions

Our data show distinct molecular subtypes among high-risk non-*BRCA1/BRCA2* tumours based on somatic mutational signatures including (1) tumours with high HRDetect score explained by methylation in *BRCA1* or mutations in other HRD-genes, (2) tumours with high mutational burden and low HRDetect score, (3) mutationally quiescent tumours with low HRDetect score, no RS 2 signature but often *CDH1* mutations, and (4) mutationally quiet tumours with low HRDetect score but high RS 2 signature and no *CDH1* mutations.

Further larger studies are demanded to validate these findings. The identified substructure among the mutationally quiescent non-*BRCA1/BRCA2* tumours may point to common aetiological mechanisms within the subgroups. Critically, whatever these unknown factors are, they clearly drive the increased risk of carcinogenesis through other pathophysiological mechanisms than mutagenesis. The results also indicate a strong potential for the classification of variants based on mutational signatures using HRDetect. The finding of low BRCAness measured by HRDetect among non-*BRCA1/BRCA2* familial cancer indicates a low false positive rate for the classification of VUS in this clinically relevant patient group. Our results may also be relevant for future treatment decisions. A potential benefit of platinum-based chemotherapy has been reported for non-*BRCA1/BRCA2* patients with a high HRDetect score compared to those with a low HRDetect score [8]. Furthermore, breast cancer patients with *BRCA1/BRCA2*-deficiency have recently been shown to benefit from PARP inhibitor treatment [9] which is well-known in ovarian cancer, where *BRCA1/BRCA2* mutational status is routinely used for directing this treatment [29, 30].

## Materials and methods

### Patient material

In this study, 23 non-*BRCA1/BRCA2* patients from families with a strong history of breast cancer, previously included in a study predicting BRCAness by RNA profiling [17], were selected where matched tumour and blood samples were available. Inclusion criteria to enter the study were (1) a pedigree indicating monogenic inheritance of breast cancer predisposition, (2) the presence of ovarian cancer in pedigrees with breast cancer cases, or (3) a very young age at diagnosis of breast cancer (< 30 years). Furthermore, four *BRCA1* and three *BRCA2* patients carrying a pathogenic *BRCA1/BRCA2* variant with unknown family history were selected as controls for BRCAness classification. All tumour tissues were freshly frozen primary breast tumours collected between 1982 and 2008 in Odense and had been stored in the tumour biobanks of the Department of Pathology, Odense University Hospital and Danish Breast Cancer Cooperative Group (DBCG). Data for Immunohistochemistry (IHC) of estrogen receptor (ER), progesterone receptor (PR) and human epidermal growth factor receptor 2 (HER2) status was received from DBCG. The ER, PR and HER2 hormone receptor statuses not identified by the pathological review were estimated from gene expression levels of *ESR1, PGR* and *ERBB2*. The PAM50 subtypes were also classified for all samples from the gene expression (Additional file 5: Table S1).

### Family risk from BOADICEA breast cancer estimation model

The Breast and Ovarian Analysis of Disease Incidence and Carrier Estimation Algorithm (BOADICEA) was used to validate the increased risk of breast cancer in the patients based on their family history [19]. Five patients did not show an increased risk of breast cancer according to BOADICEA but were still included due to either early-onset breast cancer, bilateral breast cancer, multiple breast or ovarian cancers in the family, or a combination of those (Additional file 1: Figure S1, Additional file 5: Table S1).

### Whole-genome sequencing (WGS)

Sample preparation was performed using Illumina TruSeq Nano protocol with 550 bp insert length to strengthen the detection of structural variants. Samples were sequenced on Illumina Novaseq 6000 with paired-end 2 × 150 bp. The average sequencing coverage was 50.2X for tumour samples and 38.5X for normal samples (Additional file 6: Table S2).

### Gene expression

Gene expression analysis was performed using a customized version of Agilent SurePrint G3 Human GE 8 × 60 K Microarray and raw data were pre-processed as previously described [18]. Microarray data have been deposited to the Gene Expression Omnibus (GSE49481).

### Alignment of WGS data

The paired-end reads resulting from the sequencing were aligned to the human reference genome (GRCh37) using BWA-MEM v0.7.17. The specific version used can be found in the cgpmap-3.0.4 docker image (https://dockstore.org/containers/quay.io/wtsicgp/dockstore-cgpmap:3.0.4).

### Processing of WGS data

The whole-genome sequencing data was processed using the same bioinformatic pipeline as in Nik-Zainal et al. [11].

CaVEMan (Cancer Variants Through Expectation Maximization: http://cancerit.github.io/CaVEMan/) was used for calling somatic and germline single nucleotide variants (SNVs). A lightly modified version of Pindel 2.0 (http://cancerit.github.io/cgpPindel/) was used for calling somatic and germline insertions and deletions (indels).

BRASS (BReakpoint AnalySiS: https://github.com/cancerit/BRASS) was used to detect rearrangements and other structural variants.

For annotation of the resulting variant calls we used the VAGrENT (Variation Annotation GENeraTor: https://github.com/cancerit/VAGrENT) annotation tool.

The Battenberg algorithm (https://github.com/cancerit/cgpBattenberg) was used for the detection of copy number variation in matched tumour-normal samples.

The specific versions of the tools used are found in the cgpwgs-2.1.0 docker image (https://dockstore.org/containers/quay.io/wtsicgp/dockstore-cgpwgs:2.1.0).

TitanCNA [31] was further used to validate LOH of the *RAD51D* c.202G > A variant in tumour 11.

### Filtering variants

#### Germline variants

Germline variants were filtered using a candidate gene list of 170 pathogenic and likely pathogenic germline variants associated with hereditary cancer [32]. Then filters were applied keeping only frameshift, splice-site, and nonsynonymous variants with strong bioinformatic prediction and with frequency < 0.01 according to gnomAD and ExAC [33]. The variants were evaluated using the variant databases ClinVar and HGMD, and six missense variant predictors were implemented in VarSeq. Loss-of-function (protein truncating) and splice variants, variants with strong bioinformatic prediction, and variants in genes associated with breast cancer risk with an odds ratio above two [34] were selected for further investigation.

#### Somatic variants

Somatic variants were filtered using the default settings of the tools in the bioinformatic pipeline. Somatic driver mutations were identified by filtering the list of somatic variants for the driver genes previously identified in 560 breast cancers using identical criteria for reporting a driver event as in [11]. Copy number variants were furthermore filtered to also include the copy number status of the genes in which germline variants were identified, such that the analysis of LOH was possible.

### Polygenic risk score

We applied the polygenic risk score with 313 SNPs (PRS_313_) developed for breast cancer risk prediction [13] incorporated in the latest version of BOADICEA [19] to predict the risk of getting breast cancer for each individual in our cohort under the assumption that they did not already develop breast cancer.

### Mutational signatures

We applied a mathematical model [12] implemented in the Signature Tools Lib R package [35] (https://github.com/Nik-Zainal-Group/signature.tools.lib) to fit substitution and rearrangement signatures imprinted in the breast cancer genomes, i.e. first a catalogue of substitutions and rearrangements was created for each sample and then fitted using bootstrap for robustness to the twelve substitution and six rearrangement signatures previously identified [11].

### Stratification of tumours using unsupervised hierarchical clustering

Unsupervised hierarchical clustering with Euclidean distance and Ward’s linkage criterion (ward.D2 in the statistical programming language R) was used to stratify the breast cancer tumours. We incorporated both substitution and rearrangement signatures in the clustering. To make the signatures comparable, we needed to normalise the signatures to correct for the fact that cancer genomes often carry more substitution than rearrangement signatures thereby giving higher weight to the rearrangement signatures in the clustering. Proportions of signatures were normalised by dividing all substitution and rearrangement signatures by the highest proportion identified in their respective mutation categories.

### BRCAness: HRDetect and our RNA classifier

The HRDetect model for detection of *BRCA1/BRCA2*-deficient tumours [7] was applied to the patient cohort. The HRDetect model incorporates information from substitution and rearrangement signatures, HRD score and deletion of microhomology and computes the probability of each tumour being *BRCA1/BRCA2*-deficient. We used the HRDetect model implemented in the Signature Tools Lib R package [35].

We included the BRCAness classification from our in-house developed RNA classifier published in an earlier study [18]. The RNA classifier has been developed to classify basal and LumB-subtype tumours, i.e. basal-like tumours can be classified as either *BRCA1*-like or non-*BRCA1*-like, and LumB-subtype tumours can be classified as either *BRCA2*-like or non-*BRCA1/BRCA2*-like. Other subtypes are not yet supported. Molecule subtypes were identified using PAM50 as previously described [17].

### Detection of promotor methylation

Detection of promotor methylation of the breast cancer predisposition genes *BRCA1* and *BRCA2* in the patients was done in an earlier study using MLPA [17].

### Tumour mutational burden

Tumour mutational burden (TMB) is generally defined as the number of somatic mutations per megabase (Mb) within the sequenced region of the tumour sample [36–39]. In this study, the sequenced area is the entire genome. However, in many comparable studies, only exome data is available and TMB analysis on exome data is generally considered the gold standard [36]. Thus, for comparability between studies, we calculated TMB as the number of somatic substitutions and indels per Mb in the coding region of the targeted territory of the Twist Human Comprehensive Exome Panel of size 36.8 Mb (https://www.twistbioscience.com/resources/data-files/twist-human-comprehensive-exome-panel-bed-files). We used the tool tmb-wgs (https://github.com/naveedishaque/wgs-tmb) for TMB calculation which uses the approach described in Chalmers et al. [38].

### Whole-genome profiles and heatmap figures

Breast cancer whole-genome profiles were created using the Signature Tools Lib R package [35] and are presented in Additional file 3: Figure S3. Heatmaps and stacked figures (Figs. 1, 2 and Additional file 1: Figure S1) were created using the ComplexHeatmap R package [40].

### Additional information about variant interpretation

We identified very few rare germline variants in known breast cancer candidate genes. In one family, a well-known pathogenic mutation in *CHEK2* [41–43] was found as well as a high PRS score resulting in a predicted lifetime risk of 57%. In another family, a missense *TP53* germline variant, previously shown to be deleterious in a functional assay [44], accompanied by a somatic second hit in *TP53* is very likely to explain the extremely early onset breast cancer at the age of 29 years. The clinical effect of mutations identified in the candidate genes *FANCD2, RAD51D, SLX4,* and *MSH6* is less clear.

These variants included loss of function variants in *CHEK2*, *FANCD2*, *RAD51D,* and *SLX4*. In addition to the deleterious variant in *CHEK2,* we identified in another patient an in-frame *CHEK2* deletion of unknown significance, c.246_260delCCAAGAACCTGAGGA previously shown to have intermediate functional impact [42]. In another family, two affected members both carried a *MSH6* missense variant of unknown significance (VUS) c.1813A > G, p.Thr605Ala, predicted probably damaging by PolyPhen 2. No MMR signatures were identified indicating that the variant might not be pathogenic.

*FANCD2* and *SLX4* are well-established Fanconi Anemia genes similar to several other breast cancer genes. Nevertheless, mutations in these genes are expected to have low penetrance for breast cancer [34, 45–49]. In combination with other genetic risk factors e.g. a high PRS this might explain the strong familial phenotype. However, the contribution from PRS estimated from BOADICEA was minor. Nevertheless, the included families had pedigrees indicative of a strong pattern of inheritance, and therefore other yet unknown genetic risk factors are likely to play a role in these families.

Our study also indicates that tumours with pathogenic mutations in *TP53* and *CHEK2*, which are associated with DNA-damage signalling and detection of double-stranded breaks, did not classify as *BRCA1/BRCA2*-deficient tumours according to both prediction models tested. This confirms findings from earlier studies [7, 50].

The only tumour (tumour 11) with a high HRDetect score and no clear *BRCA1/BRCA2* inactivating mechanism (germline variant or methylation) had a somatic VUS in *BRCA1* and a germline missense variant in *RAD51D*. The somatic *BRCA1* variant is located in exon 11 that although containing more than half of the coding region of *BRCA1*, does not contain reported pathogenic germline missense mutations. Low allele frequency and a high copy-number level in the *BRCA1* region indicated that functional wildtype alleles exist. The variant is therefore unlikely to be causal for the high HRDetect score. The *RAD51D* variant c.202G > A, p.(Gly68Ser) has a high variant allele frequency and is located in a region with copy-number imbalance most likely in agreement with loss of the wildtype allele. According to Battenberg analysis the ploidy in the RAD51D region was 4 of which one was wildtype. However, we found this result uncertain and therefore performed CNV analysis with an independent tool TitanCNA which found clear loss of the wildtype allele (Additional file 9: Table S5). This variant has previously been reported to inactivate an ESE element and cause complete loss of full-length transcript [16]. In this study, minigene assay showed 26.7% of transcript with exon 3 skipping and 41.1% missing exon 3–5, both predicted to result in a frameshift. However, 32.2% of the transcripts had an in-frame deletion of 12 amino acids. Since it is unknown if the resulting protein is functional, the variant was classified as having unknown significance [16]. Our results strongly indicate the variant to be deleterious for the protein and therefore likely pathogenic. Our finding of low BRCAness measured by HRDetect among non-BRCA1/BRCA2 familial cancer indicates a low false positive rate for classification of VUS in this clinically relevant patient group.

## Supplementary Information

**Additional file 1. Fig. S1**: Description: Extended version of Figure 1 with information on BOADICEA family risk and polygenetic risk score.

**Additional file 2. Figure S2**: Description: The landscape of somatic mutations in whole-genome sequenced tumours.

**Additional file 3. Figure S3**: Description: Circos plots of breast cancer whole-genome profiles.

**Additional file 4. Figure S4**: Description: Distribution of mutational signature contributions.

**Additional file 5. Table S1**: Description: Patient information

**Additional file 6. Table S2**: Description: Whole-genome sequencing metrics

**Additional file 7. Table S3**: Description: Landscape of somatic mutations, per mutation type

**Additional file 8. Table S4**: Description: HRDetect Scores

**Additional file 9. Table S5**: Description: Identified somatic drivers

**Additional file 10. Table S6**: Description: Identified germline variants

## Data Availability

The datasets generated and/or analysed during the current study are not publicly available since the publication of individual genome sequencing data is not approved by the Danish Data Protection Agency for this study.
