# Supplementary material for: Non-BRCA1/BRCA2 high-risk familial breast cancers are not associated with a high prevalence of BRCAness
Source: Breast Cancer Res. 2023 Jun 14;25:69. doi: 10.1186/s13058-023-01655-y (PMC10265777; doi:10.1186/s13058-023-01655-y)
Supplement: Supplementary file 1 — Additional file 1. Fig. S1: Description: Extended version of Figure 1 with information on BOADICEA family risk and polygenetic risk score. [file 13058_2023_1655_MOESM1_ESM.docx]

**Figure S1**

**Figure S1. BOADICEA Family Risk and Polygenetic Risk Score (PRS).** 23 non-BRCA1/2 cases, four BRCA1 positive carriers (tumours 40-43), and three BRCA2 positive carriers (tumours 45-47). Patients are ordered according to PRS score. a) Germline variants possibly contribute to increased breast cancer family risk. Variants are included if they are reported pathogenic/likely pathogenic in ClinVar and the gene exists in our candidate list. Variants of unknown significance (VUS) further required a high correlation with breast cancer risk to be included (online methods). b) Age at diagnosis. c) Predicted BOADICEA family lifetime risk of getting breast cancer. d) Predicted lifetime risk of breast cancer (20-80 years) by PRS score. e) Combined family risk and PRS. The dashed line represents the mean lifetime risk (0-79 years) of breast cancer in the Danish population of 11.7% (36).
