# Supplementary material for: Non-BRCA1/BRCA2 high-risk familial breast cancers are not associated with a high prevalence of BRCAness
Source: Breast Cancer Res. 2023 Jun 14;25:69. doi: 10.1186/s13058-023-01655-y (PMC10265777; doi:10.1186/s13058-023-01655-y)
Supplement: Supplementary file 2 — Additional file 2. Figure S2: Description: The landscape of somatic mutations in whole-genome sequenced tumours. [file 13058_2023_1655_MOESM2_ESM.docx]

**Figure S2**

**Figure S2.** **The Landscape of Somatic Mutations.** The landscape of somatic mutations in 23 non-BRCA1/BRCA2 familial breast cancers, four BRCA1 positive carriers (tumours 40-43) and three BRCA2 positive carriers (tumours 45-47) ordered by HRDetect score, and BRCA1/BRCA2 positive carriers are ordered by BRCA status. a) Clinical and mutational annotation for each sample. b) BRCAness predictions: HRDetect prediction score (scores above 0.7 considered BRCAness) and RNA classifier predictions. c) Somatic substitution and indel driver mutations d) Number of substitutions in each breast cancer genome, categorised by the type of mutation. e) Number of rearrangements, categorised by rearrangement type: Deletion, duplication, inversion and translocation. f) Number of indels, subcategorised in deletion of microhomology, deletion repeat, other types of deletion, insertion and complex indels.
