# Supplementary material for: Non-BRCA1/BRCA2 high-risk familial breast cancers are not associated with a high prevalence of BRCAness
Source: Breast Cancer Res. 2023 Jun 14;25:69. doi: 10.1186/s13058-023-01655-y (PMC10265777; doi:10.1186/s13058-023-01655-y)
Supplement: Supplementary file 3 — Additional file 3. Figure S3: Description: Circos plots of breast cancer whole-genome profiles. [file 13058_2023_1655_MOESM3_ESM.docx]

**Figure S3**


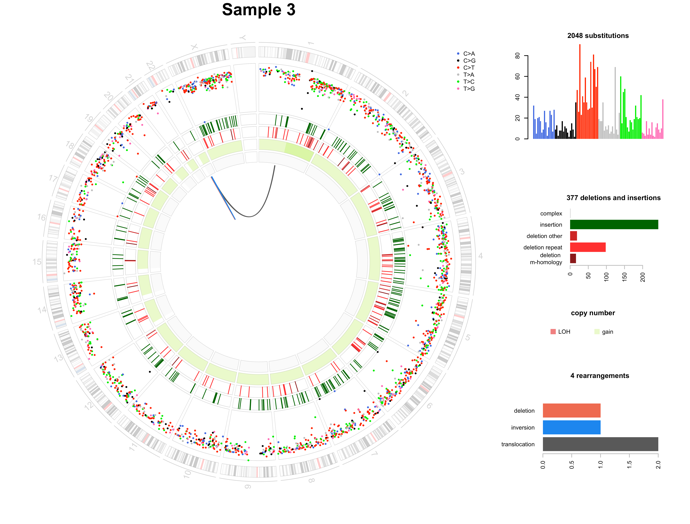

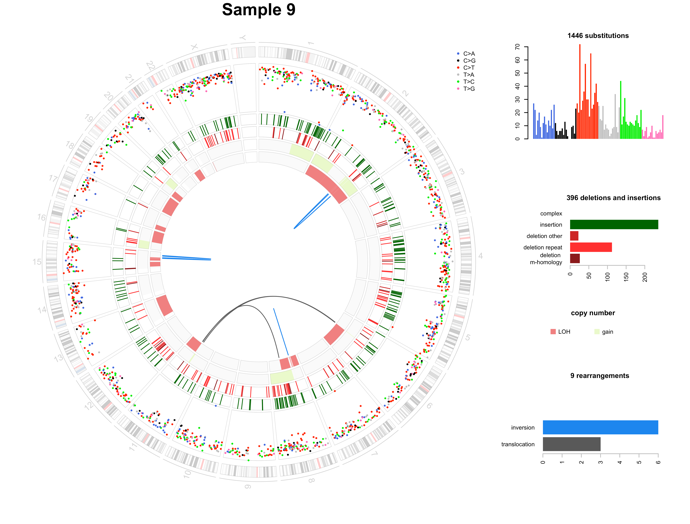

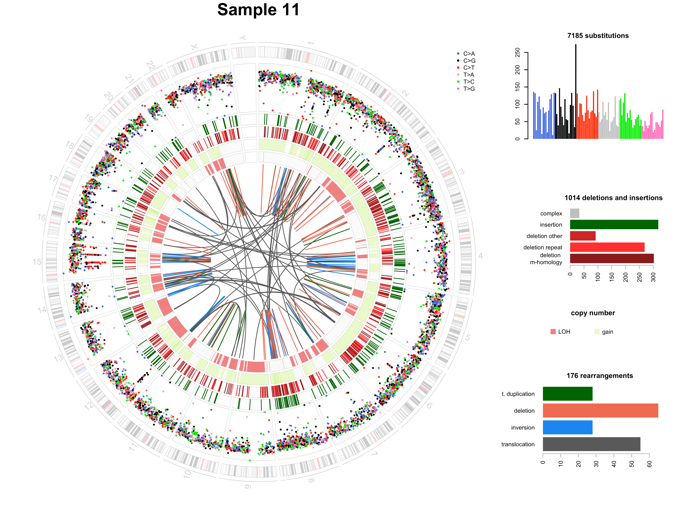

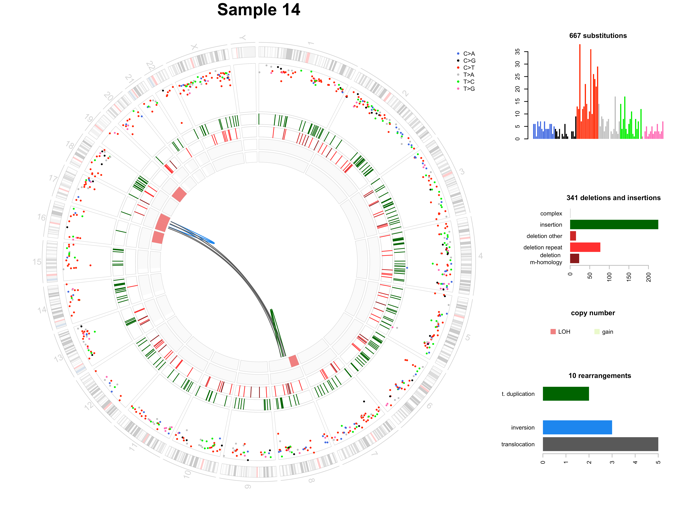

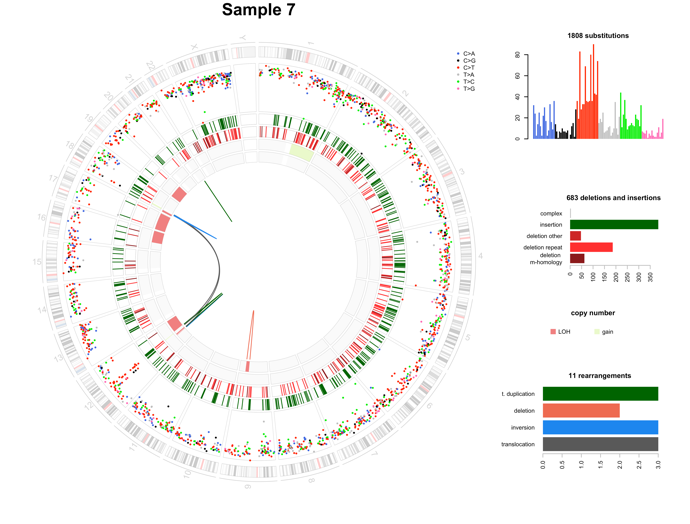

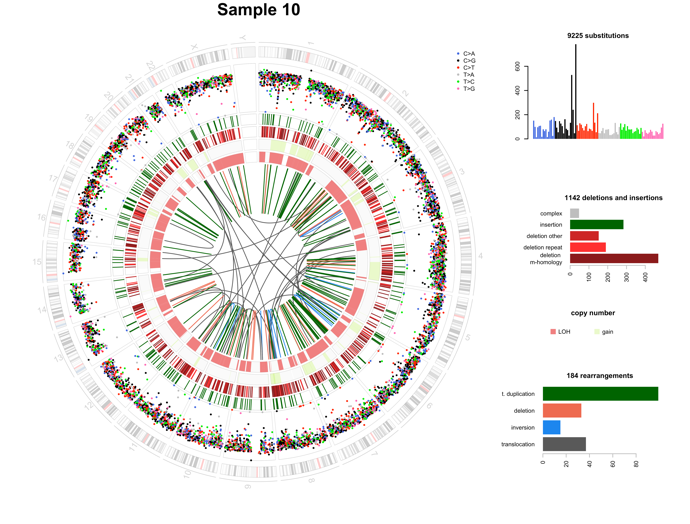

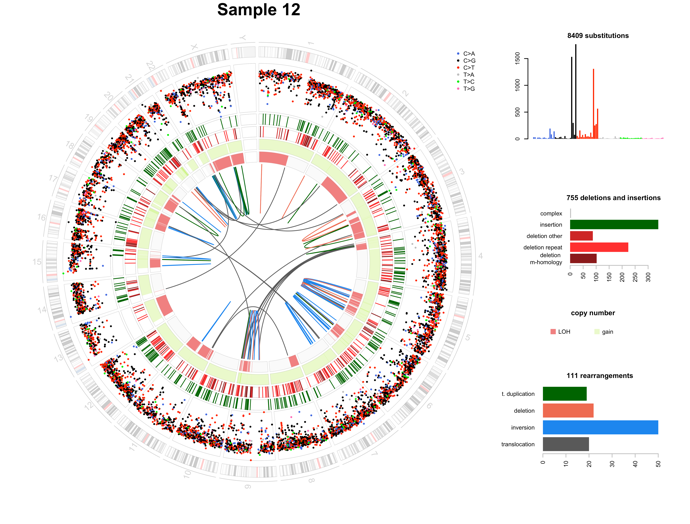

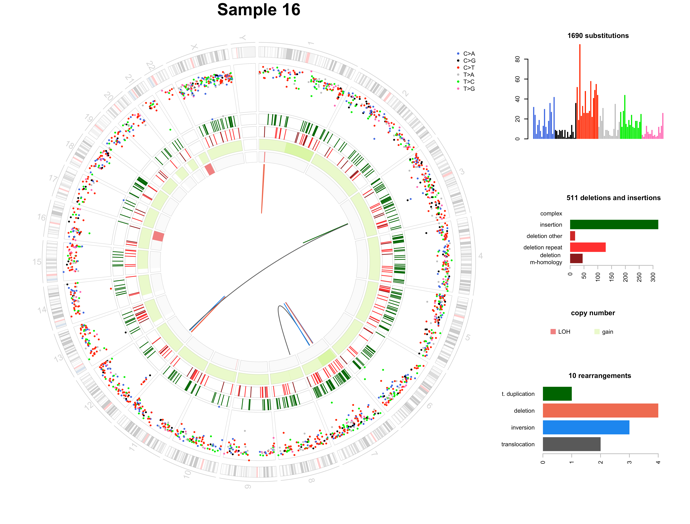

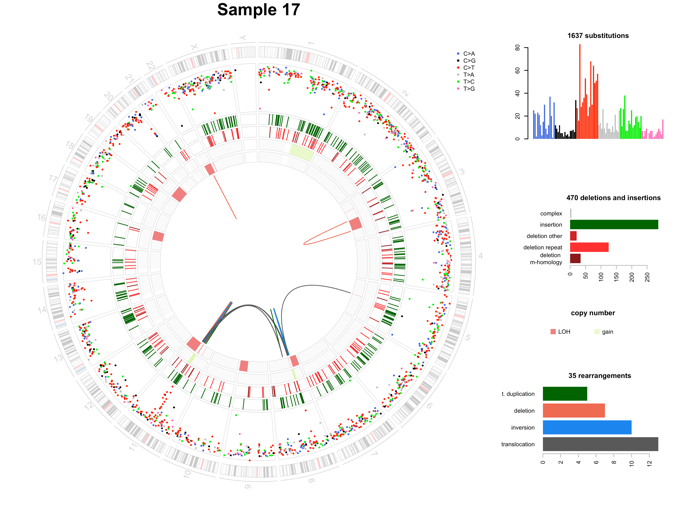

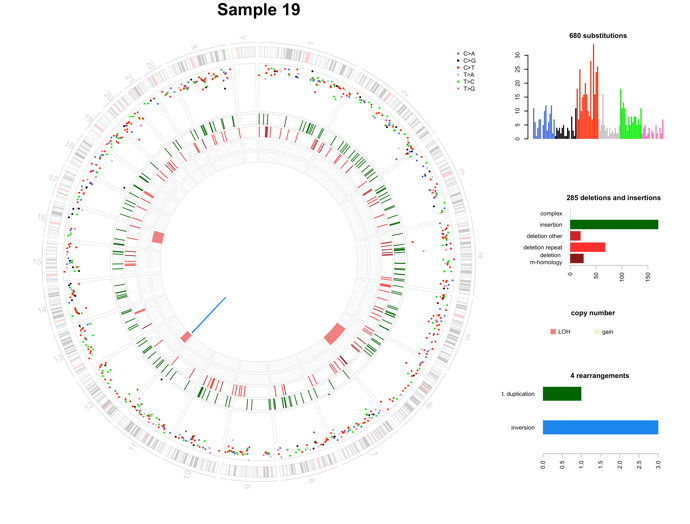

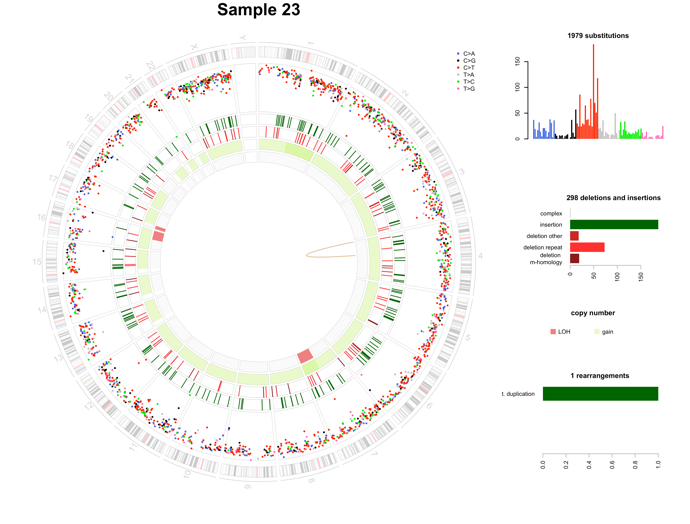

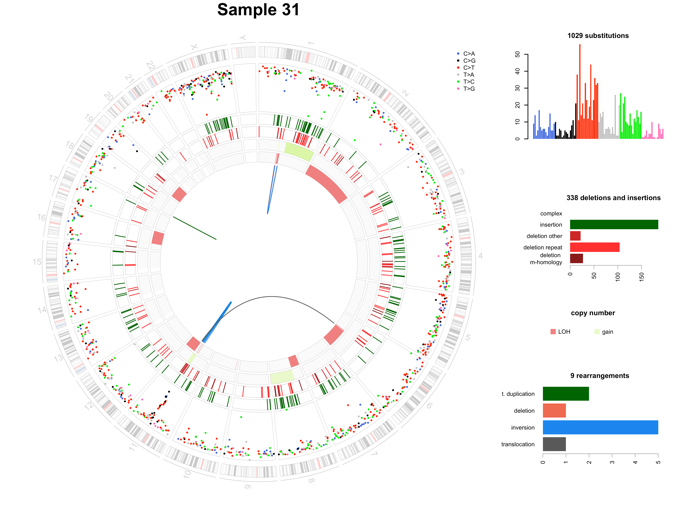

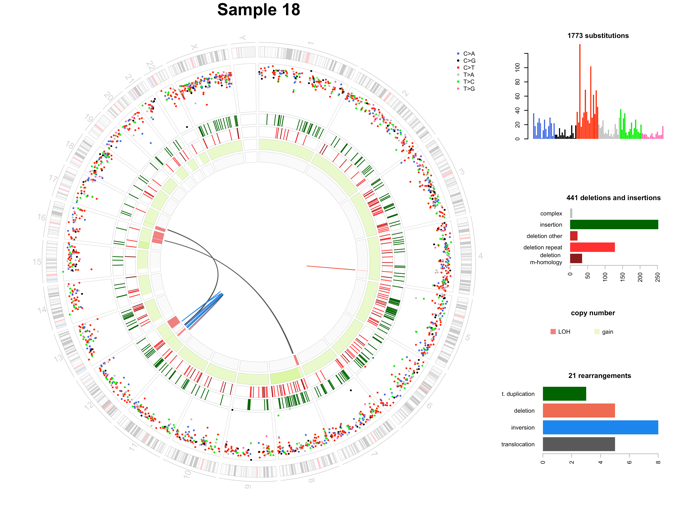

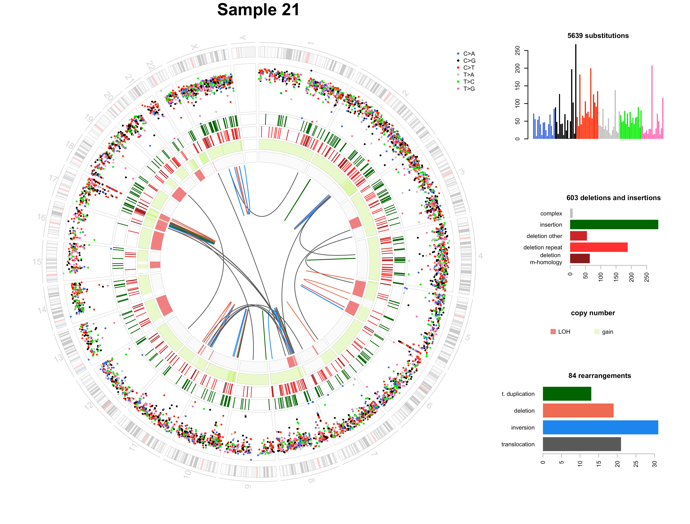

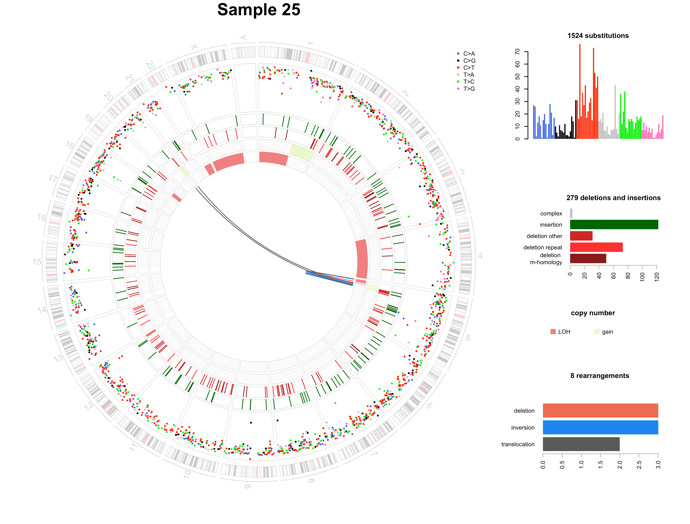

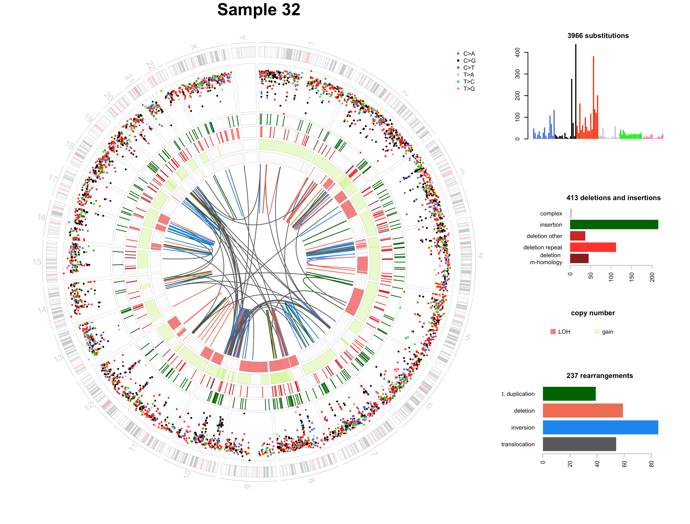

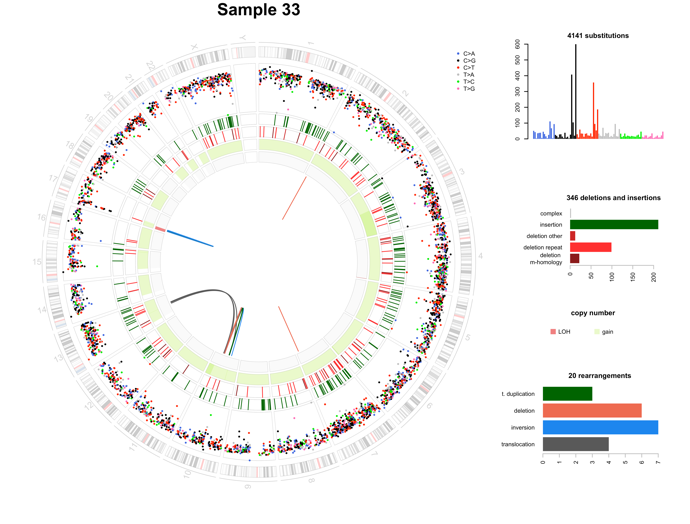

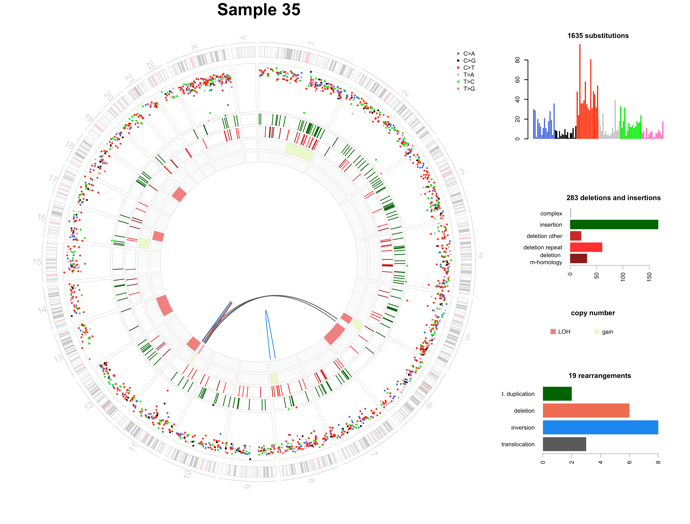

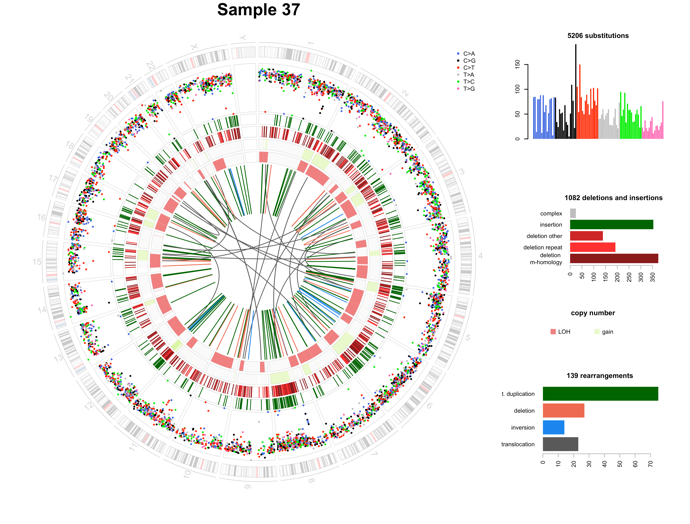

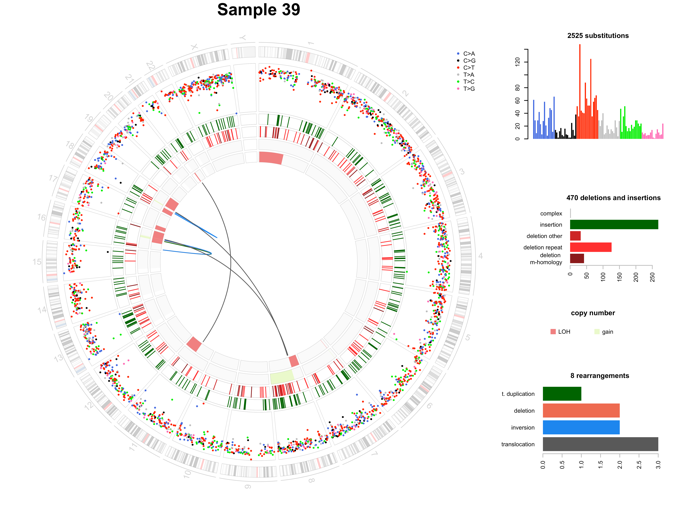

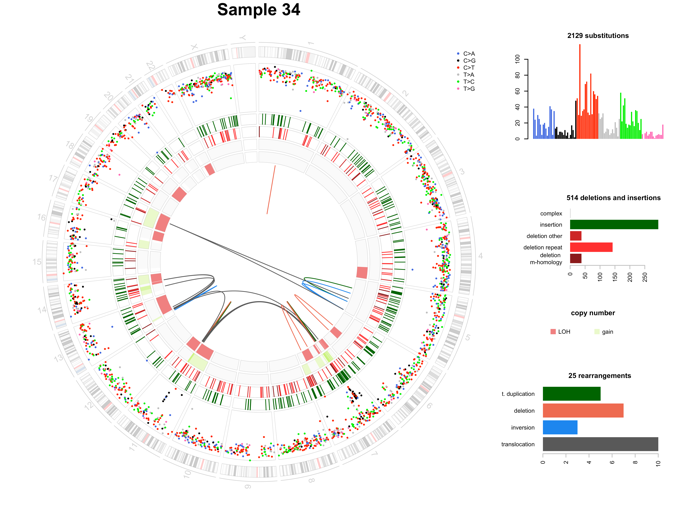

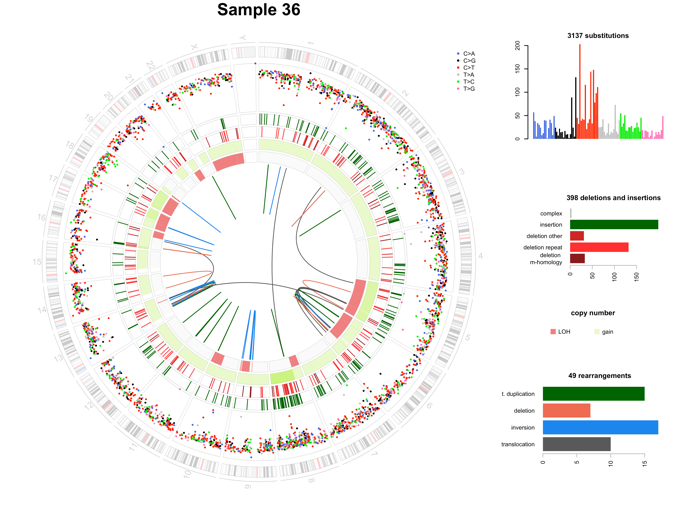

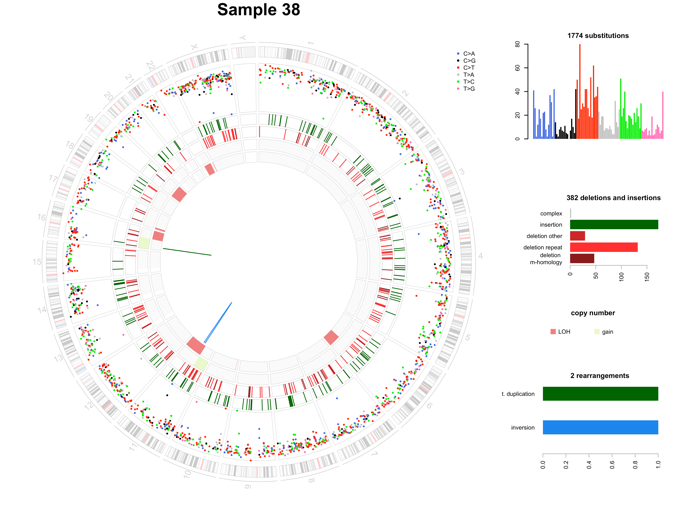

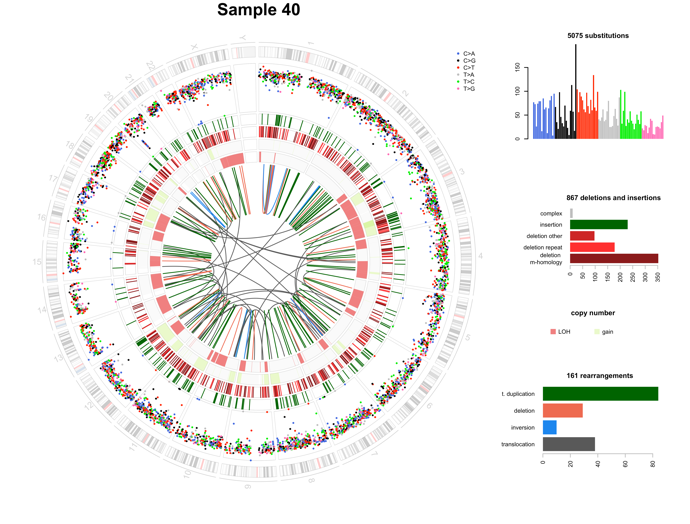

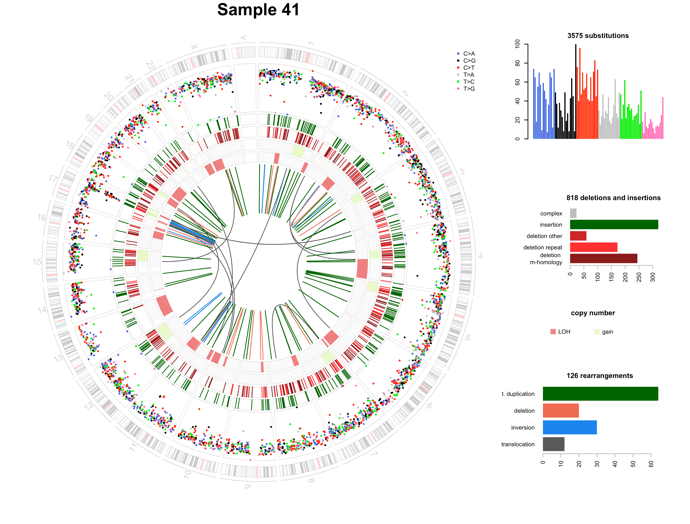

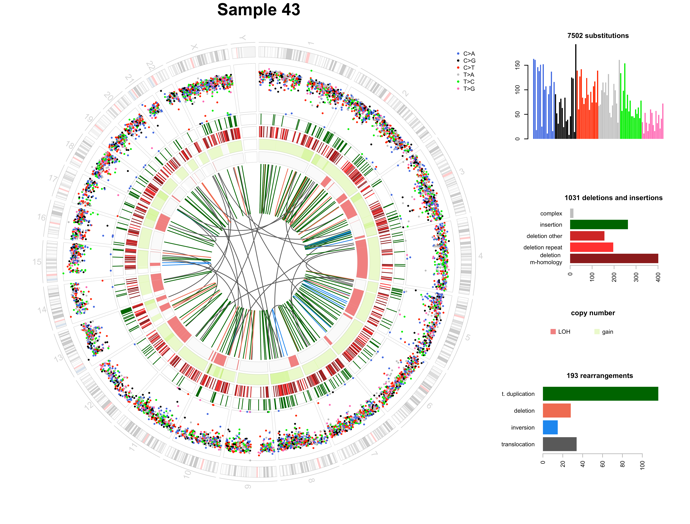

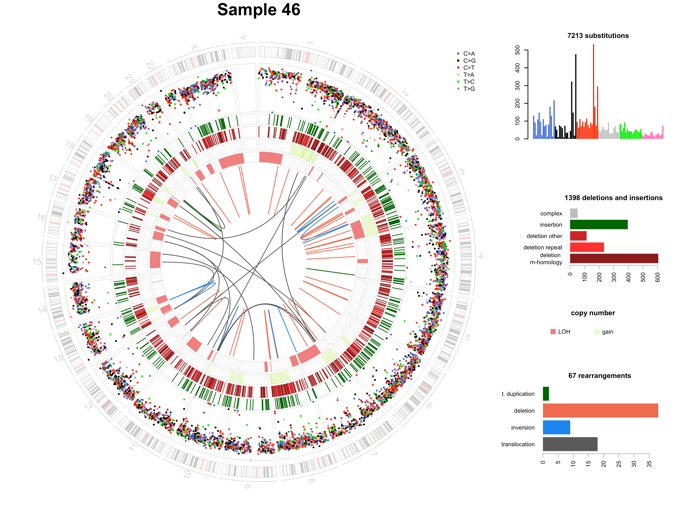


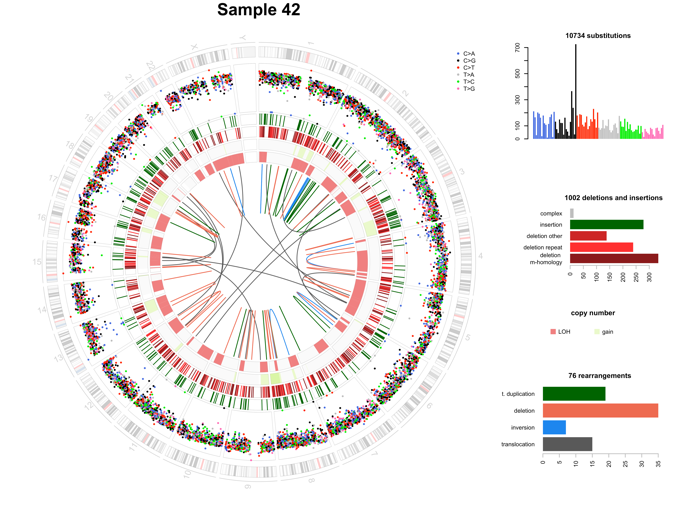

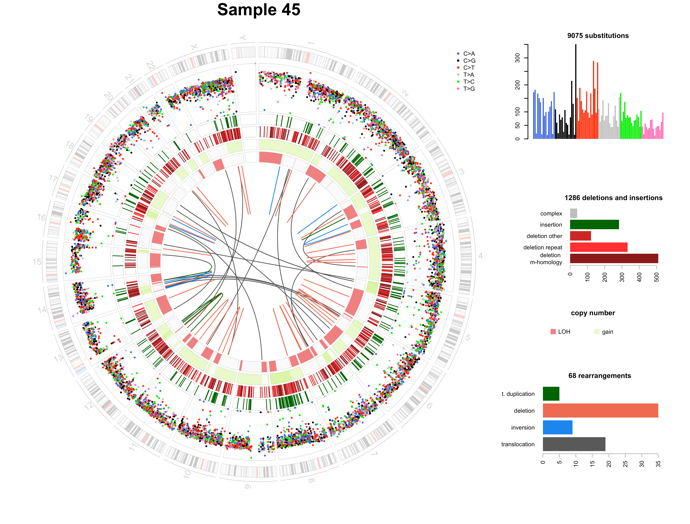

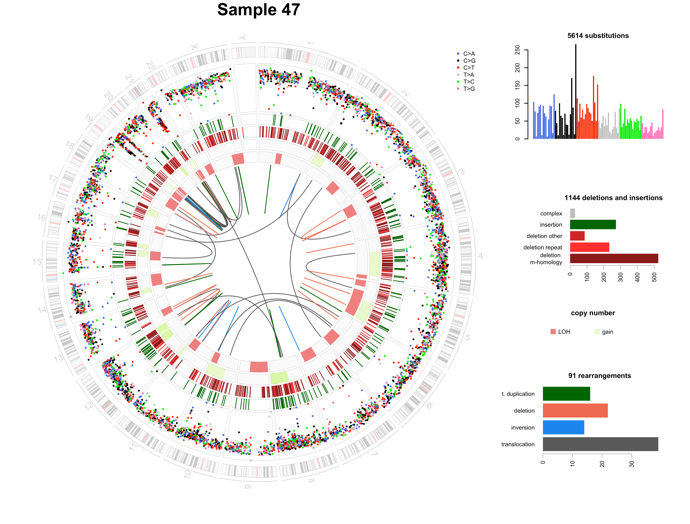


**Figure S3. Breast Cancer Whole-genome Profiles.** The circus plots depict the following features: Chromosome ideograms are shown in the outermost ring. Followed by base substitutions, presented as a rainfall plot (point colours: blue, C>A; black, C>G; red, C>T; grey, T>A; green, T>C; pink, T>G, and log10 scale of inter-mutation distances). Insertions are plotted as short green lines in the next ring, and the following ring holds deletions plotted as short red lines. The next two rings contain copy number variation, green segments represent gain and red segments represent loss. In the middle are lines representing rearrangements (green: tandem duplications, pink: deletion, blue: inversions, grey: translocations). For each individual profile, four panels are displayed on the right. From the top: the number of mutations contributing to each mutation signature fitted. The top-middle panel shows a histogram of indels. The bottom-middle panel show a legend of copy number colourings. The bottom panel shows a histogram of rearrangements.
