# Supplementary material for: Non-BRCA1/BRCA2 high-risk familial breast cancers are not associated with a high prevalence of BRCAness
Source: Breast Cancer Res. 2023 Jun 14;25:69. doi: 10.1186/s13058-023-01655-y (PMC10265777; doi:10.1186/s13058-023-01655-y)
Supplement: Supplementary file 4 — Additional file 4. Figure S4: Description: Distribution of mutational signature contributions. [file 13058_2023_1655_MOESM4_ESM.docx]

**Figure S4**

**Figure S4. Distribution of Mutational Signature Contributions.** Distribution of mutational signature contributions clustered using Euclidean distance and ward linkage criterium. Tumour names shown in bold are predicted BRCAness by HRDetect. A) shows the distribution of substitution signature contributions. B) shows the distribution of rearrangement signature contributions.
